# Effect of matrine in MAC-T cells and their transcriptome analysis: A basic study

**DOI:** 10.1371/journal.pone.0280905

**Published:** 2023-01-27

**Authors:** Zhao Zhang, Yuze Yang, Lijiao Yan, Xuerui Wan, Kangyongjie Sun, Huitian Gou, Jucai Ding, Jie Peng, Guo Liu, Chuan Wang

**Affiliations:** 1 College of Veterinary Medicine, Gansu Agricultural University, Lanzhou, China; 2 Beijing Animal Husbandry Station, Beijing, China; Ningxia University, CHINA

## Abstract

Matrine, an alkaloid derived from herbal medicine, has a wide range of biological activities, including antibacterial. Matrine was toxic to multiple cells at high concentrations. Bovine mammary epithelial cells (MAC-T) could be used as model cells for cow breast. Matrine was a feasible option to replace antibiotics in the prevention or treatment of mastitis against the background of prohibiting antibiotics, but the safe concentration of matrine on MAC-T cells and the mechanism of action for matrine at different concentrations were still unclear. In this study, different concentrations of matrine (0.5, 1, 1.5, 2, 2.5 and 3 mg/mL) were used to treat MAC-T cells for various time periods (4, 8, 12, 16 and 24 h) and measure their lactic dehydrogenase (LDH). And then the optimal doses (2 mg/mL) were chosen to detect the apoptosis at various time periods by flow cytometry and transcriptome analysis was performed between the control and 2 mg/mL matrine-treated MAC-T cells for 8 hours. The results showed that matrine was not cytotoxic at 0.5 mg/mL, but it was cytotoxic at 1~3 mg/mL. In addition, matrine induced apoptosis in MAC-T cells at 2 mg/mL and the proportion of apoptosis cells increases with time by flow cytometry. RNA-seq analysis identified 1645 DEGs, 676 of which were expressed up-regulated and 969 were expressed down-regulated. The Kyoto Encyclopedia of Genes and Genomes (KEGG) analysis indicated the following pathways were linked to matrine-induced toxicity and apoptosis, including cytokine-cytokine receptor interaction pathway, viral protein interaction with cytokine and cytokine receptor, P53 and PPAR pathway. We found 7 DEGs associated with matrine toxicity and apoptosis. This study would provide a basis for the safety of matrine in the prevention or treatment of mastitis.

## Introduction

Matrine is an alkaloid isolated from Sophora flavescens and Sophora tonkinensis, and other plants. The research showed that matrine could significantly reduce mortality in mice infected with human enterovirus 71 [1]. In a porcine reproductive and respiratory syndrome virus (PRRSV) and porcine circovirus type 2 (PCV2) co-infected mouse model, matrine interfered with viral replication and reduced damage caused by early viral infection [2]. Because of its cytotoxic effects on insect cells and induction of apoptosis, matrine had insecticidal characteristics [3]. Matrine had anticancer activity, inhibiting proliferation and inducing apoptosis and autophagy in breast cancer cells [4]. Matrine inhibited cancer cell proliferation and induced apoptosis in acute myeloid leukemia cells and hepatocellular carcinoma cells [5, 6]. In addition, matrine could also inhibit the growth and metastasis of bladder cancer cells [7]. Matrine inhibited the growth of *Escherichia coli* and *Staphylococcus aureus* [8]. Matrine inhibited growth and biofilm formation in *Staphylococcus epidermidis* [9]. Meanwhile, matrine was cytotoxic to Mouse Liver Natural cytotoxic T cells (NCTC) at medium and high concentrations, inhibiting cell viability and inducing apoptosis [10]. The high concentration of matrine could activate oxidative stress, damage mitochondria and induce apoptosis in Human Normal Liver Cells (HL-7702) [11]. Matrine was neurotoxic to zebrafish embryos/larvae at low concentrations and was lethal and teratogenic at high concentrations in zebrafish embryos/larvae development [12]. After intraperitoneal injection of half lethal dose of matrine, the mice showed neurotoxicity and liver necrosis [13]. Dairy cow mastitis is mainly caused by *Streptococcus agalactiae*, *Staphylococcus aureus* and *Escherichia coli* [14]. MAC-T cells are an artificially modified bovine mammary epithelial cell line that can serve as a model cell for cow mastitis [15]. Matrine as a candidate substitute for antibiotics was a feasible choice because of its biological activity for antibacterial against the background of antibiotic restriction in cow mastitis, but its safe dose on MAC-T cells and the functional mechanisms at different concentrations still remained unclear. Therefore, this study investigated the effects of different concentrations of matrine on MAC-T cells, screened the concentration of matrine that induces apoptosis and expected to obtain its transcriptome data, which could provide medication guidance for the application of matrine in mastitis.

## Materials and methods

### Cell cultures

Immortalized bovine mammary epithelial cell lines (MAC-T cells, 46 generations, provided by the laboratory of Veterinary Microbiology and Immunology, Gansu Agricultural University, China) were cultured in high glucose Dulbecco’s modified Eagle’s medium (DMEM, HyClone, USA) and supplemented with 10% fetal bovine serum (FBS, BI, Israel) and 1% penicillin and streptomycin solution at 37°C in a humidified atmosphere with 5% CO_2_. No sodium pyruvate DMEM (HyClone, USA) was the backup.

### Cell treatment

Matrine (Sigma, America) is a solid powder. Matrine media with final concentrations of 0.5 mg/ml, 1 mg/ml, 1.5 mg/ml, 2 mg/ml, 2.5 mg/ml, and 3 mg/ml were prepared with matrine and medium (No sodium pyruvate DMEM, 10% FBS, 1% penicillin/streptomycin), and filtered through a 0.22 μm filter to remove bacteria. The cells (48 generations)were inoculated into 96-well plates at a density of 1.5 × 10^4^ cells/well and cultured in medium (DMEM, 10% FBS, 1% penicillin/streptomycin) for 24 h. The original medium was removed when the MAC-T monolayers reached 90% confluence, and then the supplementation of matrine media with 0.5, 1, 1.5, 2, 2.5 and 3 mg/mL was separately added to the cells and incubated for 4, 8, 12, 16 and 24 h, and matrine-free DMEM was used as control.

### Activity of LDH in MAC-T after matrine treatment

The cell culture medium of each well in the previous step was harvested and centrifuged for 5 min at 5000 rpm to extract supernatant. The activity of LDH in the supernatant was evaluated using the LDH kit (Nanjing Jiancheng, Nanjing, China).

### Flow cytometry analysis in MAC-T after matrine treatment

The cells (48 generations) were inoculated at a density of 4 × 10^5^ cells per well in 6-well plates and the culture medium was cleared after 24 h. The 6-well plates were subsequently added with 2 mg/mL matrine medium and incubated at 37°C for 4, 8, 12, 16 and 24 h. The control cells were not treated with matrine. And then the cells were washed successively 3 times with PBS (Solarbio, Beijing, China), trypsinized and then centrifuged at 300 g for 5 min at 4°C. After that, the cells were mixed with 100 μL binding buffer and stained with 5 μL of annexin V-fluorescein isothiocyanate (FITC) and 5 μL of propidium iodide (PI) (Bioscience, Shanghai, China) in the dark for 15 min at room temperature. A flow cytometer (BD LSRFortessaTM Cell Analyzer, 2010 BD, USA) was used to assess cell apoptosis.

### RNA extraction, library preparation and RNA-seq

Total RNA for 8h was extracted from untreated MAC-T cells and MAC-T cells (49 generations) treated with 2 mg/mL matrine using the Ultrapure RNA Kit (ComWin, Beijing, China). Total RNA was used as input material for the RNA sample preparations. Briefly, mRNA was purified from total RNA by using poly-T oligo-attached magnetic beads. Fragmentation was carried out using divalent cations under elevated temperature in First Strand Synthesis Reaction Buffer (5X). First strand cDNA was synthesized using random hexamer primer and M-MuLV Reverse Transcriptase, then use RNaseH to degrade the RNA. Second strand cDNA synthesis was subsequently performed using DNA polymerase I and dNTP. Remaining overhangs were converted into blunt ends via exonuclease/polymerase activities. After adenylation of 3’ ends of DNA fragments, Adaptor with hairpin loop structure were ligated to prepare for hybridization. In order to select cDNA fragments of preferentially 370~420 bp in length, the library fragments were purified with AMPure XP system (Beckman Coulter, Beverly, USA). Then PCR amplification, the PCR product was purified by AMPure XP beads, and the library was finally obtained. RNA sequencing was performed by Illumina NovaSeq 6000 (Illumina Inc., San Diego, CA).

### Data processing and differentially expressed genes analysis

The raw transcriptome data were stored in the NCBI BioProject, and the accession number is PRJNA779580. The index of the reference genome (Bos_taurus_Ensemble_104) was built using Hisat2 (version 2.0.5) and paired-end clean reads were aligned to the reference genome using Hisat2. The reads numbers mapped to each gene were counted with the feature Counts V1.5.0-P3 and the FPKM values were calculated. The differential expression analysis of two groups was performed using the DESeq2 R package (1.20.0). The *P* values were adj sted using the Benjamini & Hochberg’s approach. The two levels of multiple of difference (|log2FoldChange|> = 1) and significance level (*P* value< = 0.05) were set as the threshold for screening the differentially expressed genes (DEGs).

### GO, KEGG enrichment, and protein−protein interaction (PPI) analysis of the DEGs

Gene ontology (GO) enrichment of DEGs was analyzed using the clusterProfiler R package (version 3.8.1) to correct gene length bias. GO terms with corrected *P* value<0.05 were considered significantly enriched by DEGs. The statistical enrichment of DEGs in the KEGG pathway was also tested using the clusterProfiler R package. The valuable KEGG enrichment pathway was artificially screened. The protein−protein interaction (PPI) networks (confidence score ≥ 0.40) of the DEGs were obtained using the STRING database (https://string-db.org/). The visualized PPI network of the DEGs was constructed with Cytoscape software (version 3.7.2) and screened for top 7 hub genes.

### Real-time quantitative PCR

The mRNA was reverse transcribed to cDNA using the First-Strand cDNA Synthesis kit (TransGen, Beijing, China). We performed real-time fluorescent quantitative PCR using a LightCycler96 (Roche, Switzerland) instrument. The enzyme for RT-qPCR was SYBR (Vazyme, Nanjing, China). Each reaction volume was 20 μL, including 10 μL SYBR Premix Ex Taq II (2x), 0.4 μL of upstream primer (10 mM), 0.4μL of downstream primer (10 mM), 4.0 μL of cDNA template and 5.2 μL of RNase-free ddH_2_O. The primer sequences are shown in Table 1. The procedures were as follows: 94°C for 180 s, 94°C for 10 s, 60°C for 15 s, and 35 cycles of 15 s for 72°C. β-actin was used as the internal control and the relative mRNA levels of target genes were calculated by the 2^−ΔΔCt^ method [16].

**Table 1:** The sequence of primers used in this study.

Name	Primer sequence (5’-3’)	size	NCBI No.
*β-actin*	Forward: CGGCATTCACGAAACTACT	143	AY141970.1
	Reverse: GGGCAGTGATCTCTTTCTGC		
*Caspase8*	Forward: TTCATCTGCTGCATCCTCAC	172	NM_001045970.2
	Reverse: TCTGGTACTTGTCCCCTTGG		
*TNF*	Forward: AAGCATGATCCGGGATGTGG	188	NM_173966.3
	Reverse: CACCTGGGGACTGCTCTTC		
*MYC*	Forward: GACCAGATCCCAGAGTTGGA	185	NM_001046074.2
	Reverse: TAGGCGCAAGAGTTCCGTAT		
*AKT3*	Forward: ACCGCACACGTTTCTATGGT	175	NM_001191309.1
	Reverse: TTCATGGTGGCTGCATCAGT		
*P53*	Forward: ATTTACGCGCGGAGTATTTG	174	NM_174201.2
	Reverse: CCAGTGTGATGATGGTGAGG		

### Western blotting

The cells of the Matrine-treated group (matrine 2 mg/mL, 8 h) and control group were respectively treated with the mixture of RIPA buffer (high) and a protease inhibitor (Solarbio, China) to extract total protein. The protein concentration was determined using the BCA protein assay kit (Vazyme, China). The proteins (20 mg) from each sample were separated using 12% sodium dodecyl sulfate-polyacrylamide gels (SDS-PAGE) and transferred to polyvinylidene fluoride membranes (PVDF) (GE Healthcare, Wasukesha, WI, USA). The PVDF membranes were closed with skimmed milk containing 0.05% Tween-20 (TBS-T) for 2 hours, and then incubated with anti-Smad4 (1:1,000, Proteintech Group, Inc), anti-P65 (1:1,000, Proteintech Group, Inc), anti-Caspase3 (1:1,000, Proteintech Group, Inc), anti-PrP (1:1,500, Abcam, UK), anti-BAX(1:1,000, Bioss, China), anti-Bcl-2(1:1,000, Bioss, China), anti-IL6(1:1,000, Bioss, China), anti-TNF(1:1,000, Bioss, China) overnight at 4°C. β-actin (1:1,000, Bioss, China) was the control. Next, the PVDF membranes were washed 4 times with TBST buffer, 5 minutes for the first two times and 10 minutes for the last two times, totally 30 minutes. After that, the membranes were incubated with goat anti-rabbit IgG H&L (HRP) (1:5,000, Bioss, China) for 1 hour at room temperature. Finally, the membranes were washed with TBST for 30 minutes. The protein bands were exposed by chemiluminescence (Amersham Imager 600, USA) and then analyzed by AI600 software. The grayscale values of the protein bands were analyzed using Image J (version 1.53f51) software.

### Statistical analysis

Statistical analysis was performed using the procedure of GraphPad Prism 8 (GraphPad Software Inc., USA). Data were represented as mean ±standard deviation (SD) and were evaluated by One-way analysis of variance (ANOVA). *P value<0.05 indicates a significant difference.

## Results

### Matrine induced cytotoxicity in MAC-T cells

**Fig 1:**
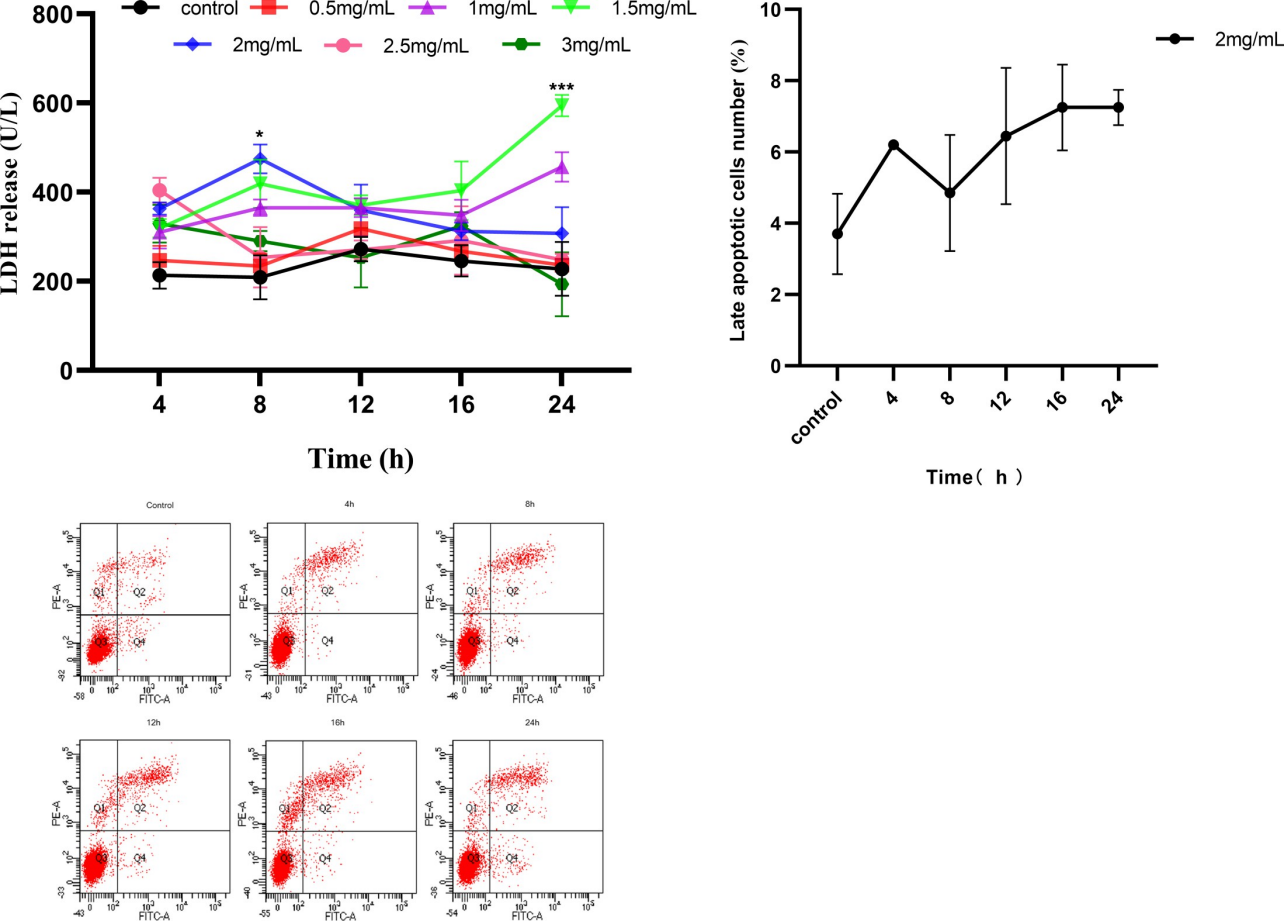
Determination of LDH release amount and apoptosis rate after treatment of MAC-T cells with matrine. A. MAC-T cells were treated with matrine at a series of concentrations (0.5-3mg/mL) for 4~24 h. The cell cytotoxicity was evaluated by the LDH assay. B. MAC-T cells were treated with matrine at a concentration of 2 mg/mL for 4~24h. Apoptosis detection with Annexin V-FITC/PI double staining in different groups by flow cytometry. The Q1 represents necrotic or mechanical damaged cells. The Q2 region represents late apoptotic cells. The Q3 region represents viable cells, while the Q4 region represents early apoptotic cells. C. Column bar graph of mean cell florescence for late apoptotic cells. The data are presented as the mean ± S.D. of three independent experiments (*p < 0.05).

The release amount of LDH can be used as an indicator of cytotoxicity. In order to study the toxicity of matrine to MAC-T cells, MAC-T cells were treated with various concentrations of matrine in order to discover its damage to MAC-T cell membrane. As shown in Fig 1A, matrine did not demonstrate cytotoxicity at low doses (0.5 mg/mL), but increased LDH release at medium and doses (1 mg/mL, 1.5mg/mL) with the increase in concentration and time, indicating that it had cytotoxicity. Surprisingly, LDH release was reduced at 2 mg/mL, 2.5mg/mL and 3 mg/mL, which might be related to matrine inhibiting the cell physiological process or inducing cell death. The cells adapted to the toxicity of matrine and developed tolerance, or matrine inhibited the physiological process of MAC-T cells and induced apoptosis. To study the effect of matrine on MAC-T cell apoptosis, we chose the best dose (2 mg/mL) of matrine for the following apoptosis experiments.

### Matrine induced apoptosis in MAC-T cells

Matrine-treated MAC-T cells were assayed with fluorescently labeled membrane-linked protein V (Annexin-V) and propidium iodide (PI). Matrine was able to induce different degrees of apoptosis in MAC-T cells. The apoptosis rate of MAC-T cells tended to increase over time, from 3.7% to 7.25% for matrine-treated MAC-T cells (Fig 1B and 1C). This indicated that matrine induced apoptosis in MAC-T cells at 2 mg/mL, and the proportion of apoptotic cells increased with time.

### Analysis of DEGs and GO term

**Fig 2:**
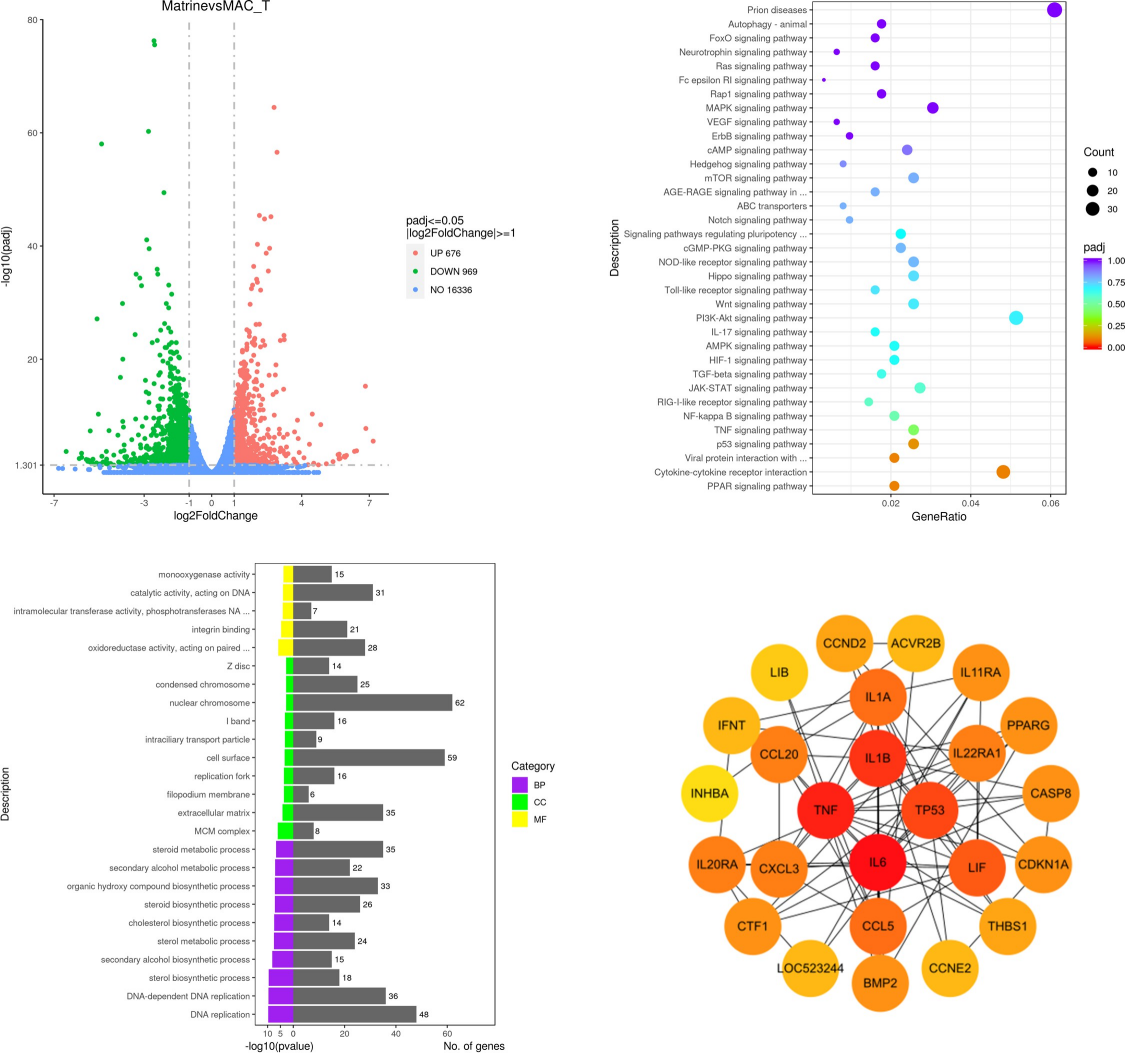
Transcriptome data analysis. A.Volcano plot of total differentially expressed genes in matrine-treated MAC-T cells. Log2|Fold Change|>1 and p< = 0.05 are thresholds. Red plots represent up-regulated DEGs and green plots represent down-regulated DEGs. B. GO functional enrichment pathway of matrine-treated groups. C. KEGG enrichment analysis of selected DEGs in matrine-treated groups. D. The protein−protein interaction (PPI) networks of selected DEGs.

To study the mRNA expression of matrine-treated MAC-T cells, we performed RNA-seq. We selected DEGs through two levels of multiple of difference (|log2 (Fold Change)|>1) and significance level (*p* value<0.05) by DESeq2. Matrine treated MAC-T cells had 1645 DEGs (S1 Table) compared with the control group, of which 969 were up-regulated and 676 were down-regulated (Fig 2A). DEGs revealed a total of 5814 enrichment GO term using GO analysis (S2 Table). The top 25 enrichment GO term were displayed in Fig 2B, and the DEGs were mainly enriched in steroid metabolic process, organic hydroxy compound biosynthetic process, DNA−dependent DNA replication, DNA replication, steroid biosynthetic process, extracellular matrix, cell surface, nuclear chromosome, condensed chromosome, oxidoreductase activity, acting on paired donors, with the incorporation or reduction of molecular oxygen, integrin binding and catalytic activity, acting on DNA.

### KEGG pathway and PPI network analysis

**Fig 3:**
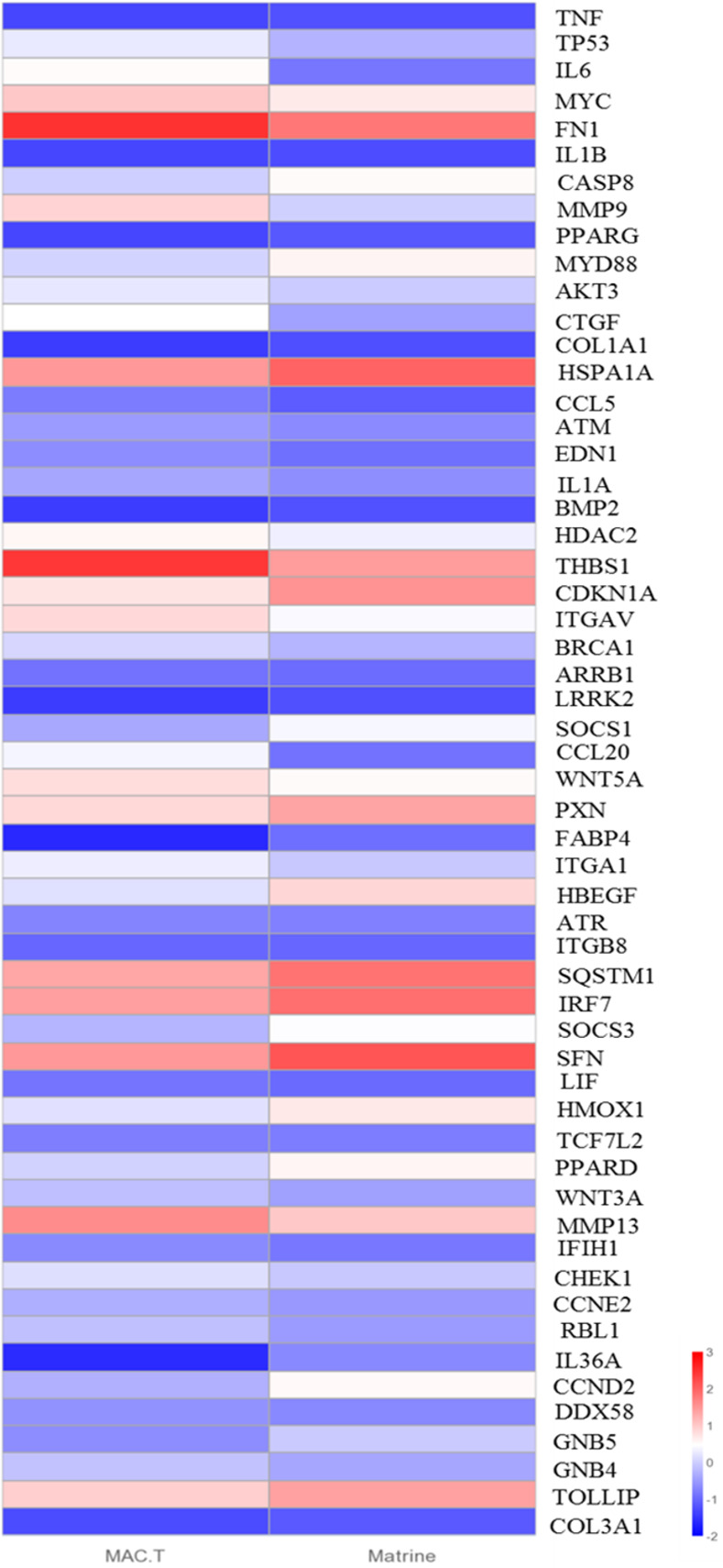
Clustering diagram of important differentially expressed genes. Red represents highly expressed genes and blue represents lowly expressed genes.

It identified 309 enrichment pathways from 1645 DEGs (S3 Table). To explore the KEGG pathway linked to matrine-induced toxicity and apoptosis, we screened 35 relevant signaling pathways from 309 KEGG pathways, including cytokine-cytokine receptor interaction, PI3K-Akt pathway, Prion diseases, MAPK pathway, JAK-STAT pathway, P53 pathway, TNF pathway, FoxO pathway, HIF-1 pathway, Rap1 pathway, cAMP pathway, Ras pathway, Toll-like receptor pathway, mTOR pathway, TGF-beta pathway and NF-κB pathway (Fig 2C). Viral protein interaction with cytokine and cytokine receptor, cytokine-cytokine receptor interaction, P53 pathway and PPAR pathway were most significant. In addition, a PPI network was created utilizing 25 DEGs from the 4 signaling pathways. The PPI networks were shown in Fig 2D, and the hub gene was screened using the Cytoscape program. In the PPI network, the hub genes in the rank of gene degree were *IL6*, *TNF*, *IL1β*, *TP53*, *LIF*, *IL1α* and *CCL5*. In the transcriptome results, the expression levels of hub genes were all decreased. These hub genes, which were related to cell proliferation, apoptosis, and inflammation, might be responsible for the toxicity of matrine. In 35 pathways, we chose DEGs with a degree greater than 10 and utilized their FPKM as the expression level to generate a heatmap (Fig 3). There were 56 DEGs in the heatmap, 37 genes of which were down-regulated and 19 genes of which were up-regulated (S4 Table).

### The validation of transcriptomic results

**Fig 4:**
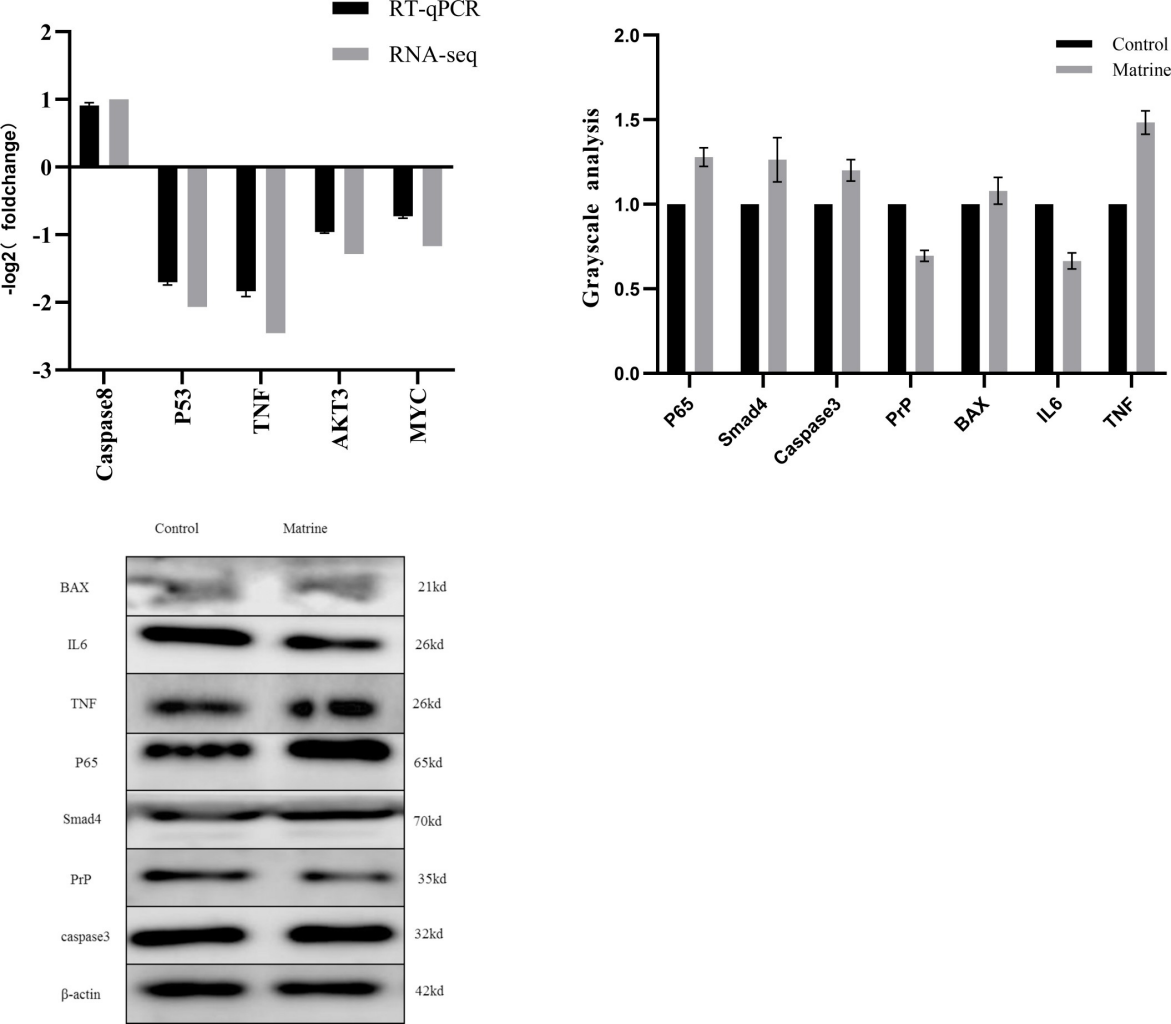
The DEGs were verified by qPCR and the effect of matrine on the expressions of apoptosis-related proteins was measured by western blotting. A. Comparison of the relative expression of DEGs screened by RT-qPCR and RNA-seq for the matrine-treated group. B. matrine-treated group immunoblots showing expression levels of BAX, IL6, TNF, Caspase3, P65, Smad4, and PrP. C. Gray-scale analysis of matrine-treated group showing expression levels of BAX, IL6, TNF, Caspase3, P65, Smad4, and PrP.

We confirmed the RNA-seq results by detecting the mRNA expression of certain genes by qRT-PCR. As shown in Fig 4A, in the matrine-treated group, the mRNA expression of *TNF*, *AKT3*, *MYC*, and *P53* were considerably down-regulated, whereas the mRNA expression of *Caspase8* was significantly up-regulated, which were consistent with the RNA-seq data.

### The expression of inflammatory and apoptotic proteins

Western Blotting results were displayed in Fig 4B and 4C, and the protein relative expression levels of TNF, P65 and Smad4 were upregulated in matrine (2 mg/mL, 8 h) group compared with those of the control group. The protein level of PrP and IL6 was down-regulated. However, the levels of apoptosis-related proteins BAX and Caspase3 were elevated compared with those of the control group.

## Discussion

Matrine-type alkaloids include asoxymatrine, sophocarpine and sophoridine [17]. Matrine, as a natural quinazoline alkaloid, has been used to treat diseases since ancient China. According to the *Compendium of Materia Medica*, matrine could clear heat, dispel evil, wind and dampness, and brighten eyes [18]. In PRRSV/PCV2 co-infected porcine alveolar macrophages, matrine inhibited PRRSV and PCV2 replication by suppressing PRRSV/PCV2-induced NF-κB pathway and the expression of TLR3 and TLR4 [19]. As an insecticide, matrinet inhibited Acetylcholinestrase activity and reduced the activity of antioxidant enzymes (SOD, CAT and POD) in insects [20]. Matrine inhibited human colon cancer cells growth and induced apoptosis by regulating the upregulation of apoptosis-related proteins Caspase3, Caspase9 and Bax/Bcl-2 ratio [21]. Matrine inhibited breast cancer cell migration by reducing the activation of MMP9/MMP2, P65, VEGFR1 and epidermal growth factor (EGF) [22]. Matrine modulated virulence genes of *Staphylococcus epidermidis*, resulting in the shedding of bacterial biofilms [23]. Matrine inhibited *Staphylococcus aureus* PVL toxin-induced apoptosis in MAC-T cells by downregulating cleaved Caspase3, cleaved Caspase8 and cleaved Caspase9 protein expression [24]. Matrine inhibited hepatocyte apoptosis and fibrosis by interfering with hepatitis B virus replication [25]. However, matrine induced apoptosis through ROS-mediated induction in normal liver cells [11]. The high concentrations of matrine were toxic to the development of zebrafish embryos/larvae, causing teratogenicity and lethality. The low concentrations of matrine produced neurotoxicity to zebrafish embryos/larvae, altering spontaneous movement and inhibiting swimming performance [12]. Matrine has been used in the clinical treatment of Infant Cytomegalovirus Hepatitis and as a common adjuvant for tumor chemotherapy drugs [26].

In this study, the high concentration of matrine (1~3 mg/mL) had a cytotoxic effect on MAC-T cells and matrine induced apoptosis at 2 mg/mL in a time-dependent manner. The results were consistent with the hepatotoxicity of matrine. LDH release amount and apoptosis increased with the increase in matrine concentrations [10]. We verified the transcriptome results via RT-qPCR, and the results were consistent with the transcriptome data. In addition, the expression of apoptosis and inflammation-related proteins was detected by western blotting. Based on the transcriptome results, we analyzed the pathways related to the toxicity and the apoptosis of matrine. The pathways related to matrine cytotoxicity included the following ones: NF-κB pathway, TNF pathway, IL-17 pathway, cytokine-cytokine receptor interaction, TGF-β pathway, mTOR pathway, Toll-like receptor pathway, Fc epsilon RI pathway, ABC transporters, PPAR pathway, Viral protein interaction with cytokine and cytokine receptor, RIG-I-like receptor pathway, HIF-1 pathway, AMPK pathway, Wnt pathway, Hippo pathway, cGMP-PKG pathway, Signaling pathways regulating the pluripotency of stem cells, Notch pathway, AGE-RAGE pathway, Hedgehog pathway, cAMP pathway, VEGF pathway, Rap1 pathway, Ras pathway, Neurotrophin pathway and Prion diseases pathway. Viral protein interaction with cytokine and cytokine receptor, cytokine-cytokine receptor interaction and PPAR pathway were most significant. In an animal model for the treatment of arthritis, matrine inhibited NF-κ B pathway, and TNF and IL-1 β levels were significantly decreased in serum by ELISA [27]. The expression of *TNF* and *IL1β* were also downregulated in this study, but TNF was upregulated by western blotting. TNF, a multifunctional proinflammatory cytokine, activated various signal transduction pathways, including NF-κB pathway, MAPK pathway, mTOR pathway, TGF-β pathway and TNF pathway [28]. TNF was involved in the regulation of cell proliferation, differentiation, inflammation, apoptosis and other biological processes [29]. IL1β belonged to the interleukin-1 family and was pro-inflammatory cytokines with agonistic activity. The inactive IL-1β precursor was hydrolyzed by Caspase1 into active IL1β, which is involved in the inflammatory response [30]. IL1α was also a member of the interleukin-1 family and was upregulated by a diverse array of inflammatory stimuli [31]. IL1β was excreted from the cell very early together with IL-1α in the course of inflammation, inducing first localized inflammation and eventually systemic inflammation [32]. The transcriptome results of this study showed decreased expression of *IL1α* and *IL1β*. Matrine inhibited pancreatic fibrosis in rats by decreasing TGF-β and Smad2 expression in the TGF-β/Smad pathway [33]. In contrast, the gene expressions of *Smad* family and *TGF-β*, in general, did not change in our study with only Smad4 protein increased. MTOR encoded phosphatidylinositol kinase-related kinases with complexes two forms, namely, mTORC1 and mTORC2. MTOR was mainly through the PI3K/Akt/mTOR pathway to regulate cell growth, cell cycle and other various physiological functions [34]. Matrine induced autophagy and apoptosis in MCF-7 cells by inhibiting the AKT/mTOR pathway, and phosphorylation levels of AKT and mTOR protein were significantly downregulated by western blotting [4]. In this study, *AKT3* expression was downregulated. However, *PRR5*, a component of mTORC2, was upregulated.

In this study, the high concentration of matrine induced apoptosis on MAC-T cells. Pathways linked to apoptosis include PI3K-Akt pathway, JAK-STAT pathway, p53 pathway, MAPK pathway, FoxO pathway, ErbB pathway, Autophagy, NOD-like receptor pathway. P53 pathway was most significant. Matrine inhibited IL-6/JAK2/STAT3 pathway at 0.5 mg/mL doses by downregulating IL-6 expression and reducing STAT3 and JAK2 phosphorylation levels, thereby inhibiting the growth of K562 cells [35]. IL6 was secreted by variety immune and non-immune cells, and induced the transcription of the inflammatory response through the interleukin 6 receptor [36, 37]. IL6 was a pleiotropic cytokine, and the complex of IL6 and IL6 receptors could induce activation of the JAK/STAT and PI3K pathways [38]. In this study, *IL6* was downregulated, but *JAK2* and *STAT3* expression remained unchanged, while the expression of *STAT* suppressors *SOCS1* and *SOCS3* was upregulated. In human glioblastoma cells, matrine induced apoptosis by inhibiting the PI3K/AKT/P27 pathway, in which the expression of AKT reduced while P27 increased, but P53 remained unchanged [39]. P53 (TP53) is an important transcription factor in P53 pathway, PI3K-Akt pathway, and MAPK pathway. P53 could exert antioxidant activities to inhibit reactive oxygen species by upregulating specific antioxidant genes in response to oxidative stress [40]. P53 participated in the initiation of autophagy to maintain the stability of intracellular environment and cell growth [41]. The expression of *AKT3* and *P53* was downregulated, but *P27* did not change in this study. LIF was a member of the interleukin-6 family of cytokines [42]. Both LIF and IL-6 signal through the shared cytokine receptor gp130 [43]. In this study, the expression of *LIF* was up-regulated. The study has shown that SOCS3 is highly up-regulated by LIF and it then closed the JAK/STAT signalling cascade, forming a negative feedback loop [44]. CCL5 belonged to the C-C chemokine family, and was a chemokine that was early expressed and involved in immune regulation and inflammatory process [45]. In this study, the expression of *CCL5* was inhibited by matrine. In conclusion, we obtained 4 significant KEGG pathways related to toxicity and apoptosis by transcriptome and obtained some hub genes affected by matrine from them.

## Conclusion

This study showed that the concentration of matrine at 0.5 mg/mL was a safe concentration and cytotoxic to cells at 1~3 mg/mL. We also found that matrine induced apoptosis at 2 mg/mL in a time-dependent manner. We conducted a preliminary investigation of matrine-induced apoptosis, which would provide a basis for the safety of matrine administration. Based on KEGG and PPI network analysis of the transcriptome data, we identified 4 signaling pathways and 7 hub genes related to matrine toxicity and apoptosis, and provided data on the mechanism of matrine-induced apoptosis.

## Supporting information

S1 Table All differentially expressed genes (DEGs) in Matrine treated MAC-T cells. (XLS)

S2 Table All GO functional pathway of matrine-treated groups. (XLS)

S3 Table All KEGG analysis of DEGs in matrine-treated groups. (XLS)

S4 Table Clustering diagram data of important DEGs. (XLSX)

S1 Raw images (ZIP)
