# Supplementary figures and images for: Effect of matrine in MAC-T cells and their transcriptome analysis: A basic study
Source: PLoS One. 2023 Jan 27;18(1):e0280905. doi: 10.1371/journal.pone.0280905 (PMC9882957; doi:10.1371/journal.pone.0280905)

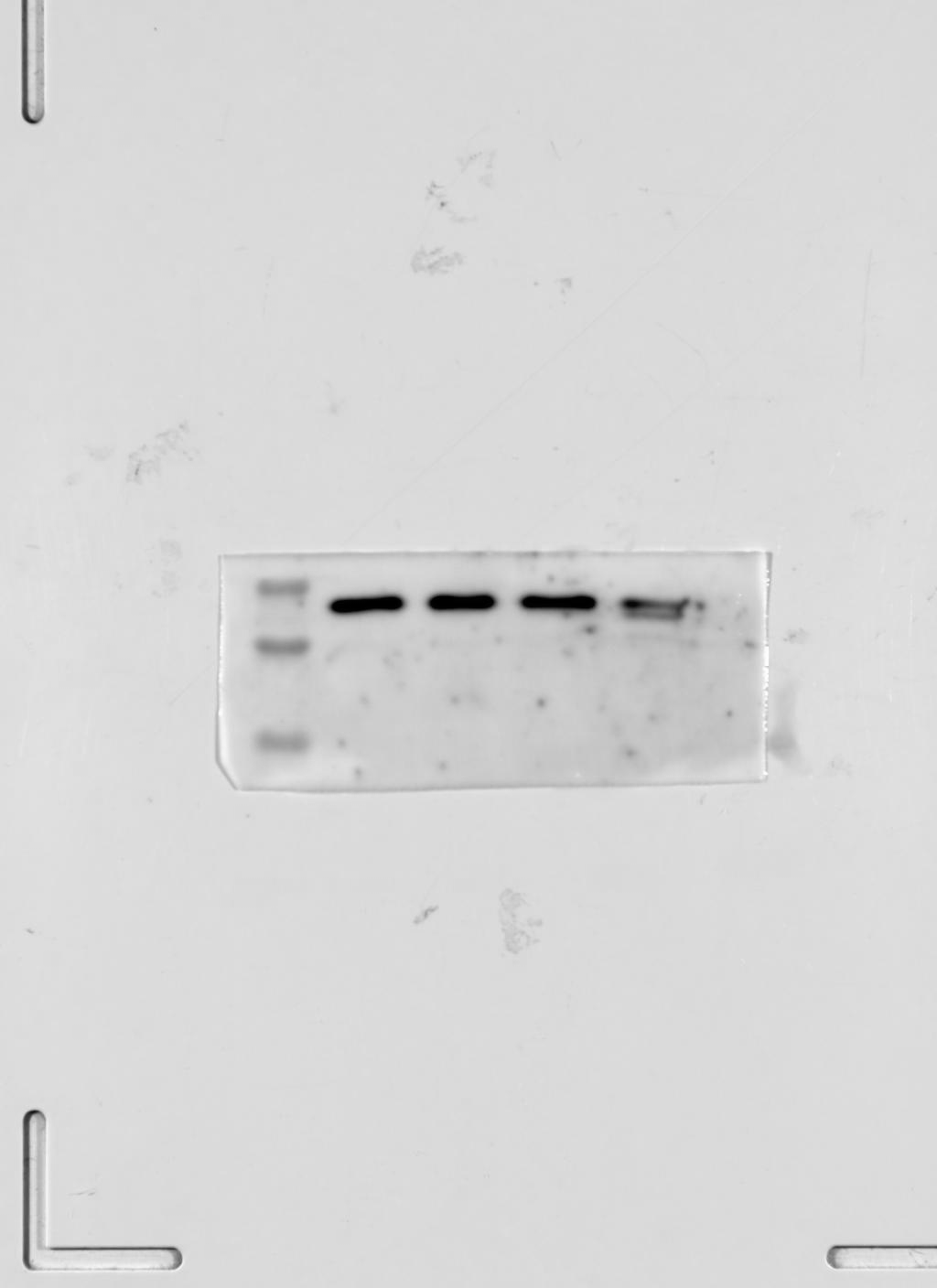

Supplement: S1 Raw images — (ZIP) [file pone.0280905.s005.zip › original raw image files in blot/CASP3+Marker.tif]

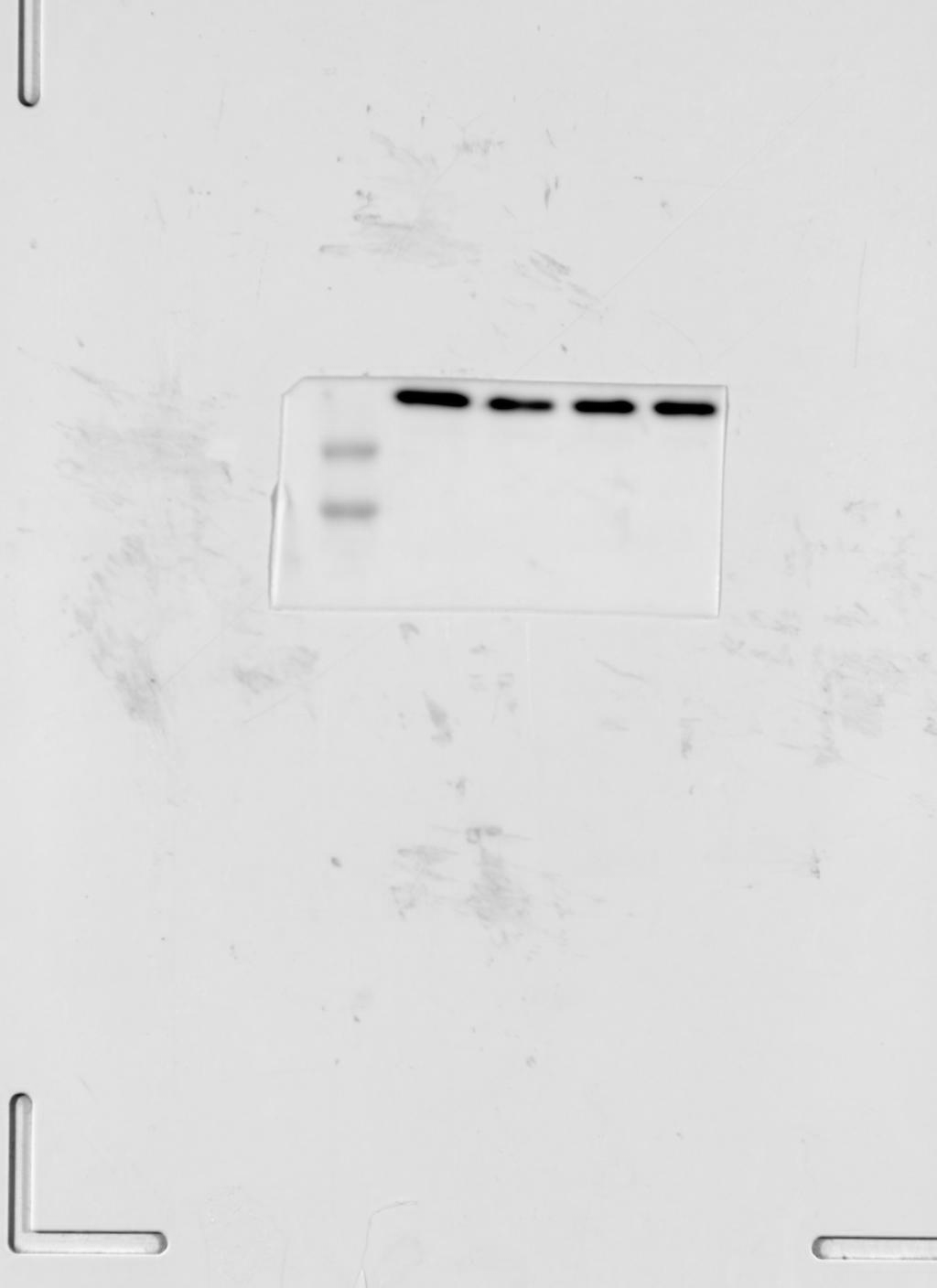

Supplement: S1 Raw images — (ZIP) [file pone.0280905.s005.zip › original raw image files in blot/IL6 +Marker.tif]

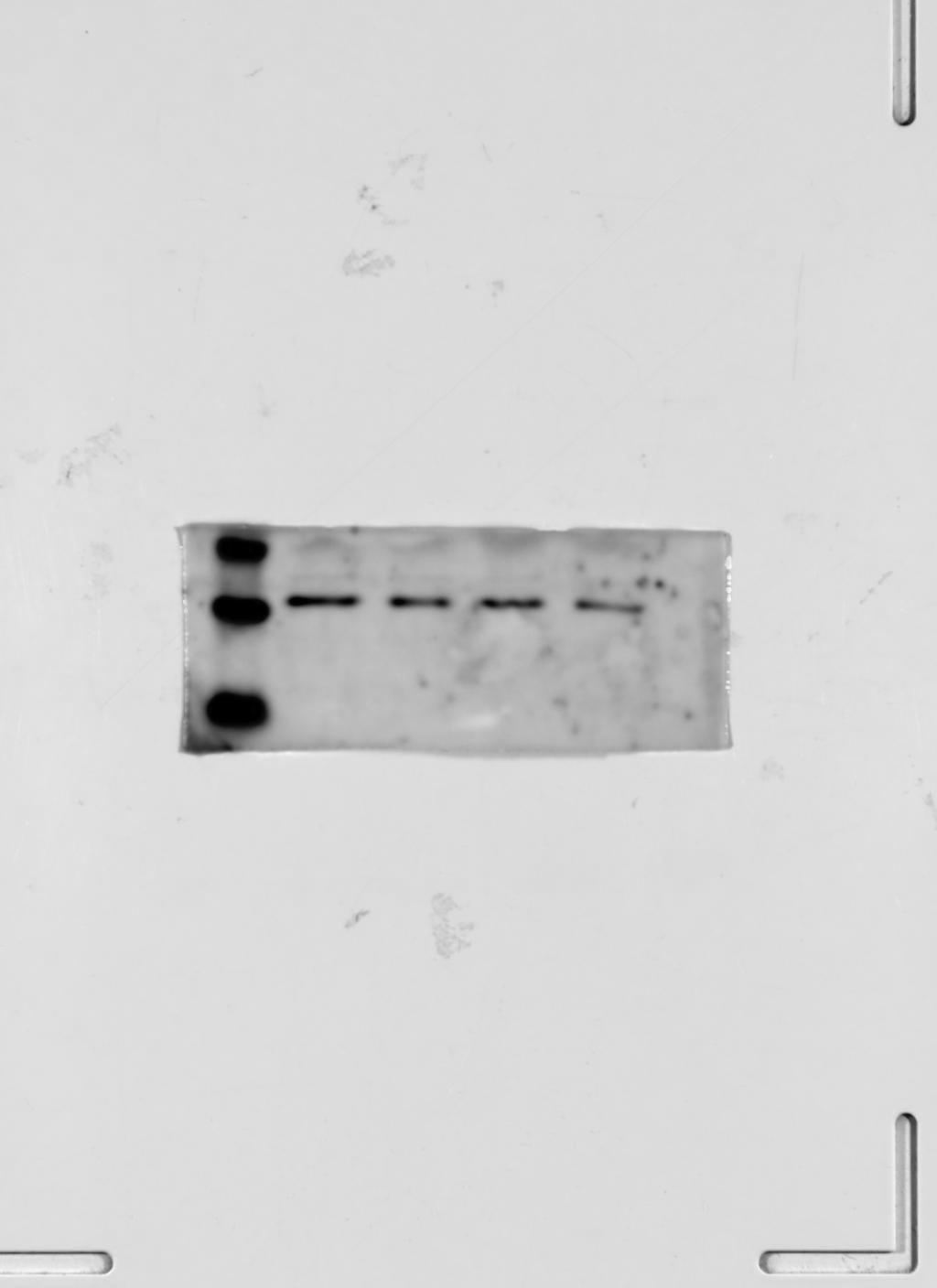

Supplement: S1 Raw images — (ZIP) [file pone.0280905.s005.zip › original raw image files in blot/PRNP+Marker.tif]

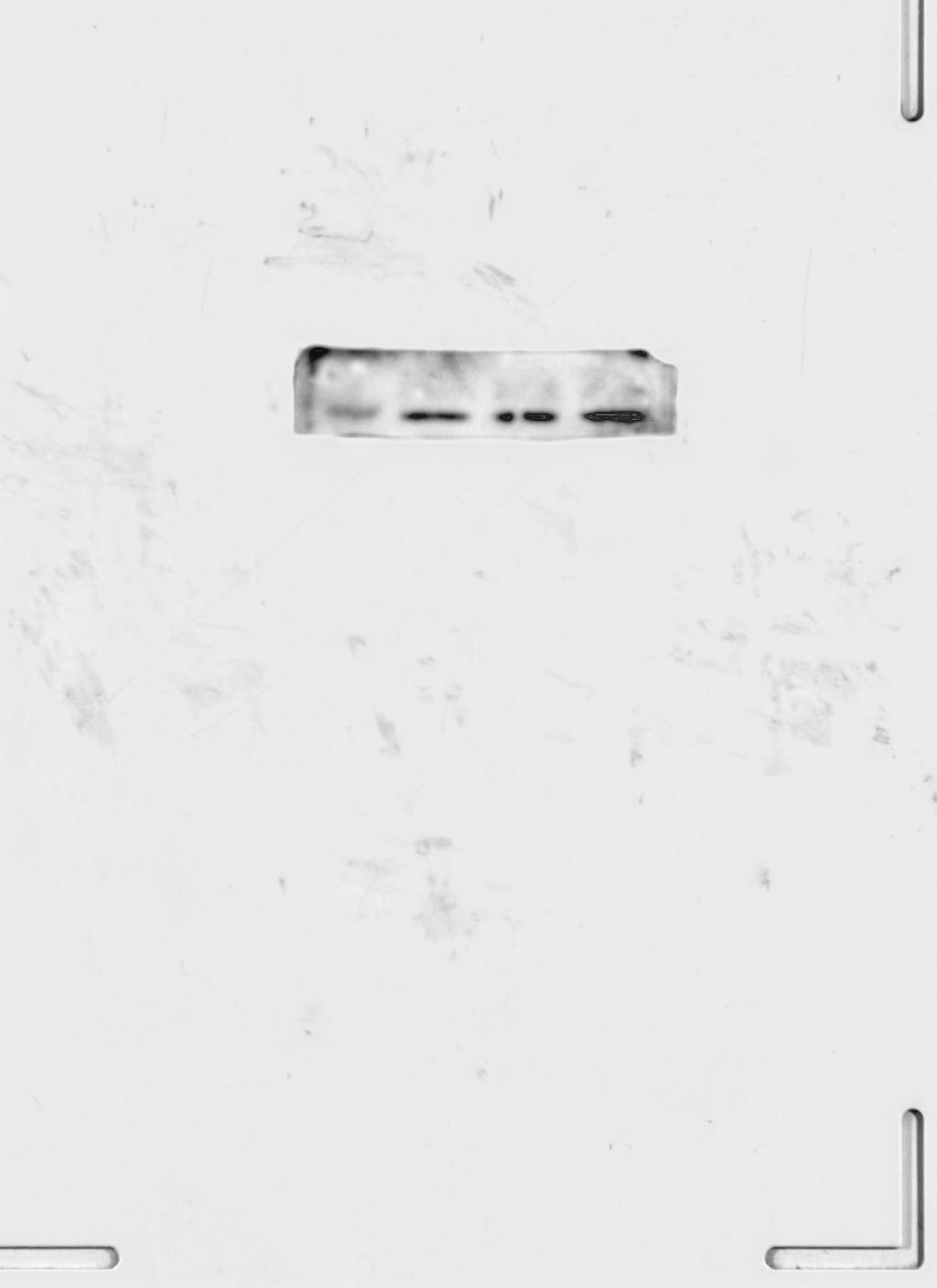

Supplement: S1 Raw images — (ZIP) [file pone.0280905.s005.zip › original raw image files in blot/TNF+Marker.tif]

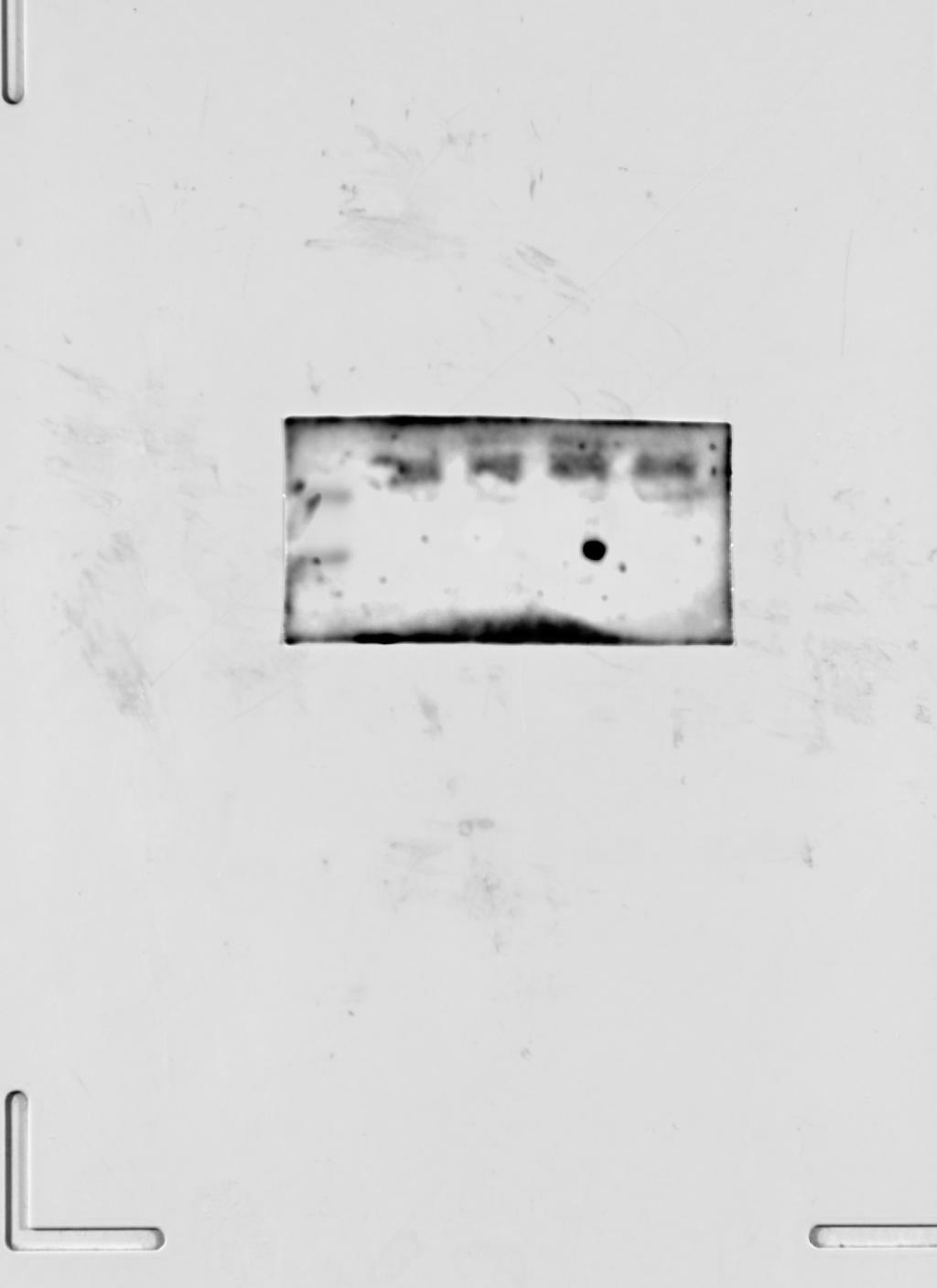

Supplement: S1 Raw images — (ZIP) [file pone.0280905.s005.zip › original raw image files in blot/bax+Marker.tif]

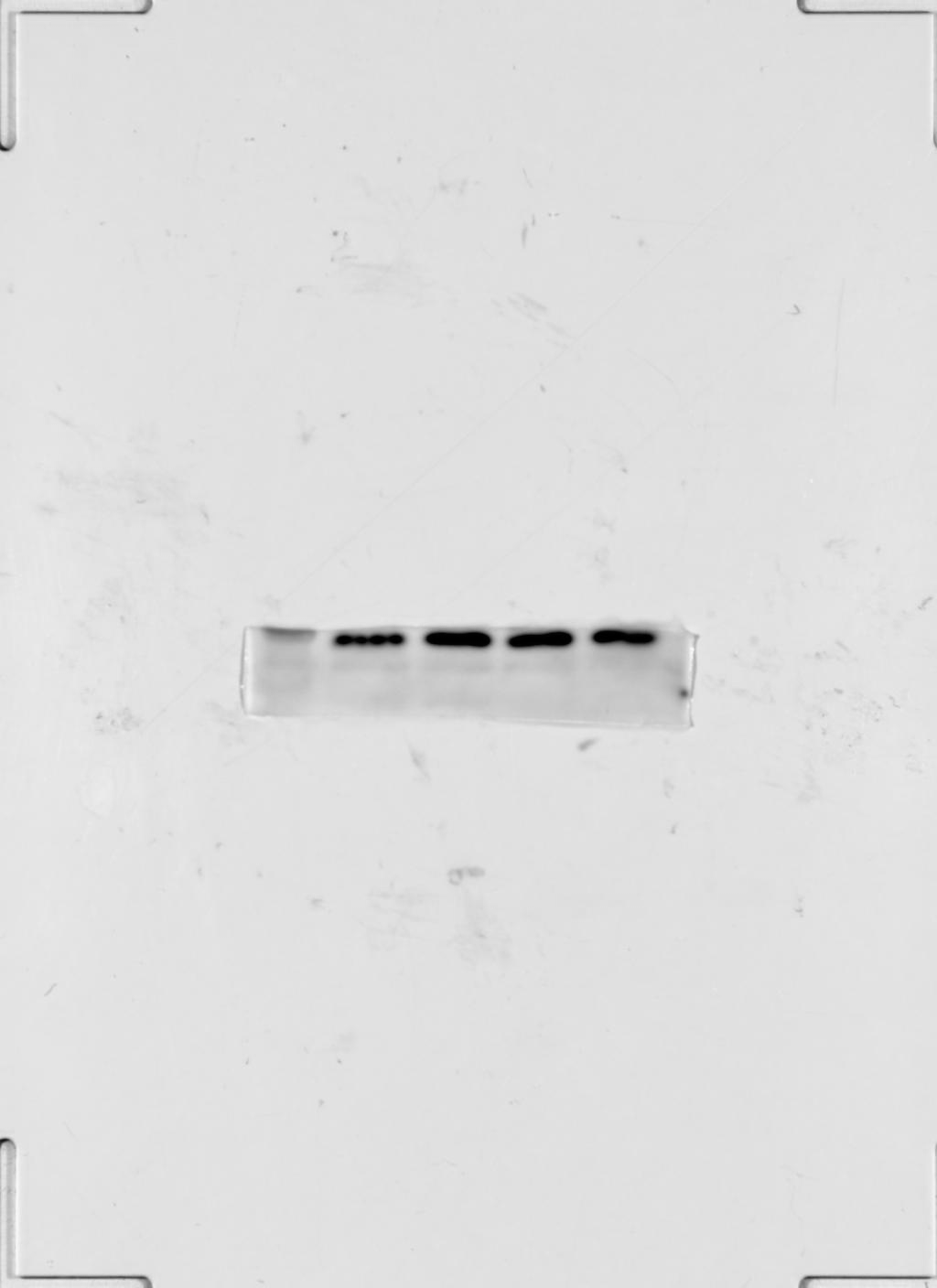

Supplement: S1 Raw images — (ZIP) [file pone.0280905.s005.zip › original raw image files in blot/p65+Marker.tif]

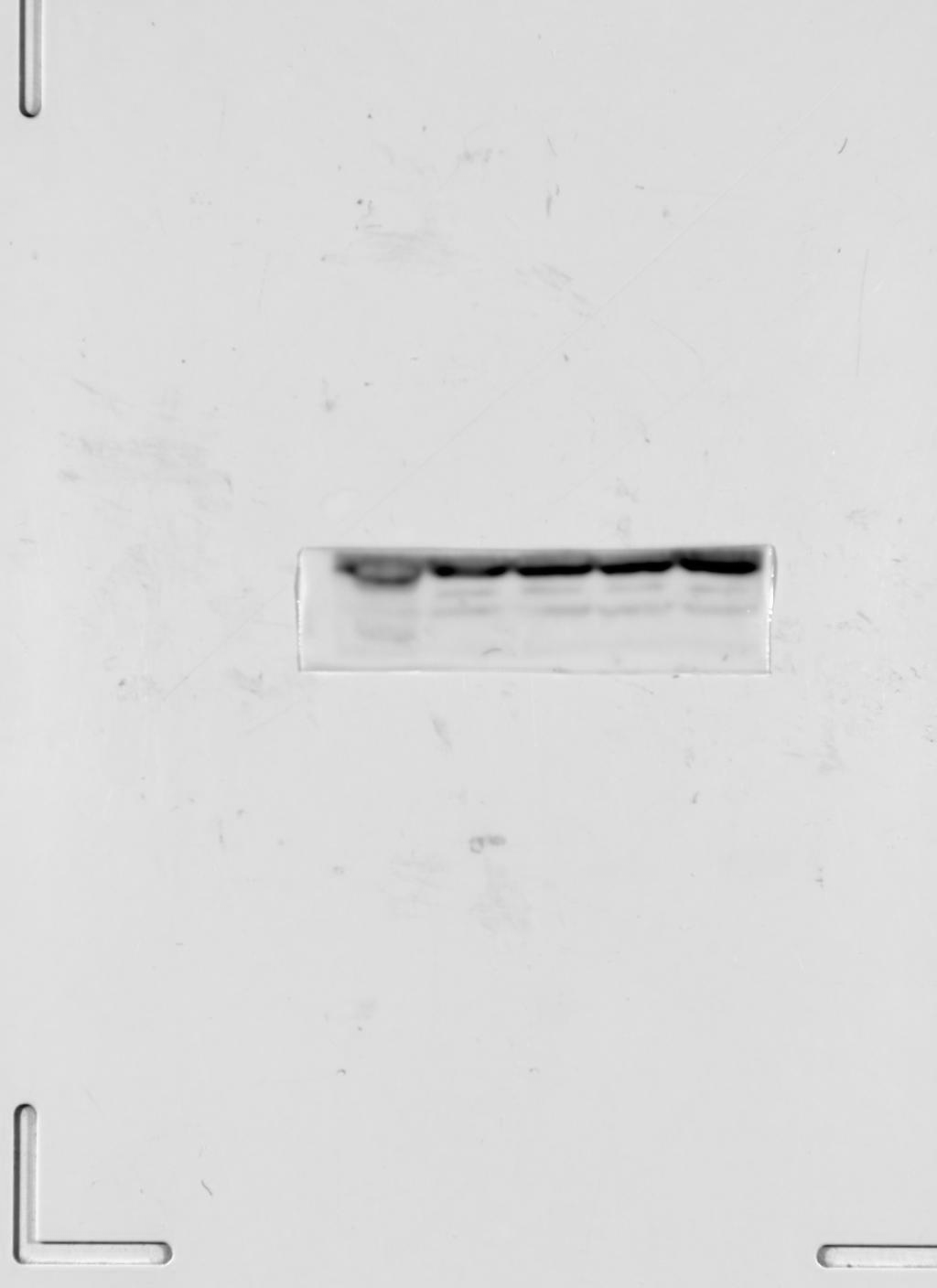

Supplement: S1 Raw images — (ZIP) [file pone.0280905.s005.zip › original raw image files in blot/zz1.19smad4-6 2022.01.19_09.16.51_Ch+Marker.tif]

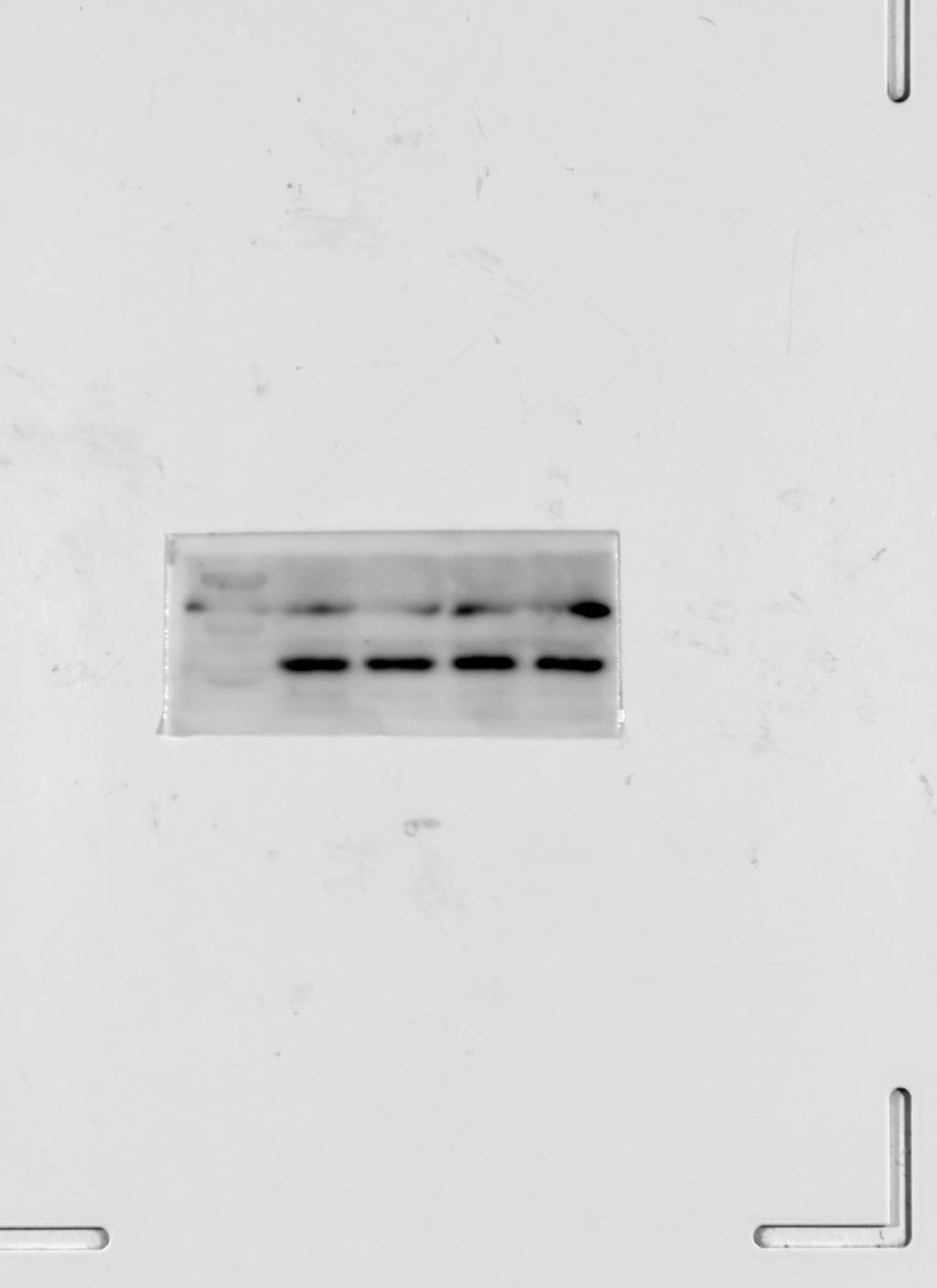

Supplement: S1 Raw images — (ZIP) [file pone.0280905.s005.zip › original raw image files in blot/a┬-actin+Marker.tif]
